# Risk of cutaneous malignant melanoma in relation to use of sunbeds: further evidence for UV-A carcinogenicity

**DOI:** 10.1054/bjoc.1999.1181

**Published:** 2000-04-03

**Authors:** J Westerdahl, C Ingvar, A Måsbäck, N Jonsson, H Olsson

**Affiliations:** Departments of Surgery, Pathology, Oncology, University Hospital, Lund, S-221 85, Sweden

**Keywords:** melanoma, ultraviolet radiation, sunbeds, risk

## Abstract

In a population-based, matched, case–control study from southern Sweden of 571 patients with a first diagnosis of cutaneous malignant melanoma and 913 healthy controls aged 16–80 years, the association between sunbed use and malignant melanoma was evaluated. A total of 250 (44%) cases and 372 (41%) controls reported ever having used sunbeds. A significantly elevated odds ratio for developing malignant melanoma after regular exposure to sunbeds was found, adjusted for hair colour, raised naevi, skin type and number of sunburns (odds ratio (OR) 1.8, 95% confidence interval (CI) 1.2–2.7). A dose–response relationship between total number of sunbed uses and melanoma risk was only found up to the level of 250 times. The OR was higher in individuals younger than age 36 years (adjusted OR 8.1, 95% CI 1.3–49.5 for regular vs never use). The association seemed to be true only for subjects with black/dark brown or light brown hair and among females. Lesions of the extremities showed the strongest association of increased risk with sunbed use. An increased risk was related to commercial exposure and to exposure during the winter. The results substantiate the hypothesis that exposure to sunbeds might increase the risk of developing malignant melanoma. © 2000 Cancer Research Campaign


British Journal of Cancer (2000) **82**(9), 1593–1599
© 2000 Cancer Research Campaign
DOI: 10.1054/ bjoc.1999.1181, available online at http://www.idealibrary.com on IDEAL®

### Risk of cutaneous malignant melanoma in relation to use of sunbeds: further evidence for UV-A carcinogenicity

**J Westerdahl[1], C Ingvar[1], A Måsbäck[2], N Jonsson[2] and H Olsson[3]**

Departments of [1]Surgery, [2]Pathology and [3]Oncology, University Hospital, S-221 85 Lund, Sweden

**Summary** In a population-based, matched, case–control study from southern Sweden of 571 patients with a first diagnosis of cutaneous malignant melanoma and 913 healthy controls aged 16–80 years, the association between sunbed use and malignant melanoma was evaluated. A total of 250 (44%) cases and 372 (41%) controls reported ever having used sunbeds. A significantly elevated odds ratio for developing malignant melanoma after regular exposure to sunbeds was found, adjusted for hair colour, raised naevi, skin type and number of sunburns (odds ratio (OR) 1.8, 95% confidence interval (CI) 1.2–2.7). A dose–response relationship between total number of sunbed uses and melanoma risk was only found up to the level of 250 times. The OR was higher in individuals younger than age 36 years (adjusted OR 8.1, 95% CI 1.3–49.5 for regular vs never use). The association seemed to be true only for subjects with black/dark brown or light brown hair and among females. Lesions of the extremities showed the strongest association of increased risk with sunbed use. An increased risk was related to commercial exposure and to exposure during the winter. The results substantiate the hypothesis that exposure to sunbeds might increase the risk of developing malignant melanoma. © 2000 Cancer Research Campaign

**Keywords**: melanoma; ultraviolet radiation; sunbeds; risk

The only established exogenous causal factor for cutaneous malignant melanoma is exposure to sunlight (IARC, 1992). It is widely believed that the ultraviolet (UV) radiation component of solar radiation is responsible for this relationship. Although the transition from solar to artificial sources of UV radiation as a potential risk factor for melanoma development is logical, only limited attention has been paid to non-solar UV radiation and melanoma risk. In addition, such studies may shed light on the effect of different wavelength ranges on the melanoma development.

Concerning use of sunbeds and/or sunlamps and risk of melanoma results so far have been somewhat inconclusive. Some studies, including a previous study from us, have pointed out a significant association between sunbed or sunlamp use and melanoma (Swerdlow et al, 1988; Walter et al, 1990; Autier et al, 1994; Westerdahl et al, 1994). In another study only limited evidence of a relation was found (Chen et al, 1998). In contrast, other investigations have not been able to demonstrate such an association (Gallagher et al, 1986; Holman et al, 1986; ∅sterlind et al, 1988*b*; MacKie et al, 1989).

We have conducted a new population-based, matched case–control study of malignant melanoma in the South Swedish Health Care Region to be able to address the issue further.

### MATERIALS AND METHODS

The study identified 709 persons, aged 16–80 years, in the South Swedish Health Care Region with a first histopathological diagnosis of cutaneous *invasive* malignant melanoma between 1 January 1995 and 30 June 1997, according to the population-based Regional Tumour Registry. The permission of the physician responsible for the treatment of each patient was sought. In two cases the physician did not respond, and in additional 33 cases the patient was considered ineligible by the treating physician (18 were ineligible for psychological reasons, four had another serious disease, three were dead, three refused to participate, two had metastases, one was found to have the wrong diagnosis, one had not been fully informed and one had moved). Thus, the case group comprised 674 eligible persons.

For each of these cases two healthy controls, matched by sex, age (within a year) and parish were selected by random sampling from the National Population Registry of residents of the South Swedish Health Care Region.

All eligible cases ($n$ = 674) were mailed a comprehensive questionnaire including different epidemiological variables (medical history, medicaments, family history, constitutional factors, educational level, UV radiation exposure, smoking habits and alcohol) within 2 months following diagnosis. During the same time all selected controls ($n$ = 1348) were mailed an identical questionnaire. Non-responders were contacted twice.

A total of 584 cases (86%) and 1028 controls (76%) answered the questionnaire. Thirteen cases with no matched control and 115 controls with no matched case were excluded. Thus, the subjects actually studied consisted of 571 cases (84% of 674 eligible cases) and 913 controls (68% of 1348 healthy controls selected).

The following information was collected with regard to sunbed use: ever exposure; regular exposure; exposure time (i.e. how long each exposure to sunbed is/was); number of times per week; number of weeks per year; location of exposure; season when exposure took place; age at first use; and age at last use. An

*Received 21 September 1999*
*Revised 25 November 1999*
*Accepted 25 November 1999*

*Correspondence to:* J Westerdahl

**Table 1** Exposure to sunbeds among controls. The estimated percentages of total number of controls belonging to a given category are given with 95% confidence intervals

| | Exposure to sunbeds | | |
|---|---|---|---|
| | **Never** | **Sometime** | **Regular (present or past)** |
| Total (*n* = 910) | 59 (56–62) | 30 (27–33) | 11 (9–13) |
| Sex | | | |
| Males (*n* = 453) | 76 (72–80) | 25 (21–29) | 8 (5–10) |
| Females (*n* = 457) | 43 (38–47) | 41 (36–45) | 16 (13–20) |
| Age | | | |
| 18–35 (*n* = 87) | 20 (11–28) | 47 (37–58) | 33 (23–43) |
| 36–60 (*n* = 381) | 45 (40–50) | 42 (37–46) | 14 (10–17) |
| 61–80 (*n* = 442) | 79 (76–83) | 16 (13–20) | 4 (3–6) |
| Hair colour | | | |
| Black/dark brown (*n* = 240) | 60 (54–66) | 27 (22–33) | 13 (9–17) |
| Light brown (*n* = 495) | 59 (54–63) | 31 (27–35) | 10 (8–13) |
| Blond/fair (*n* = 110) | 56 (46–65) | 32 (23–40) | 13 (6–19) |
| Red (*n* = 39) | 56 (41–72) | 33 (18–48) | 10 (1–20) |
| Skin reaction to sun exposure after a few days of exposure | | | |
| Tan/no burn (*n* = 118) | 58 (49–66) | 31 (23–40) | 11 (5–17) |
| Moderate tan (*n* = 479) | 54 (50–59) | 34 (30–38) | 12 (9–15) |
| Light tan (*n* = 272) | 64 (58–70) | 25 (20–31) | 11 (7–14) |
| No tan (*n* = 10) | 90 (71–100) | 10 (0–29) | 0 |
| Number of raised naevi | | | |
| None (*n* = 770) | 63 (60–67) | 27 (24–30) | 10 (8–12) |
| 1–2 (*n* = 100) | 37 (28–46) | 42 (32–52) | 21 (13–29) |
| > 2 (*n* = 40) | 35 (20–50) | 55 (40–70) | 10 (1–19) |
| Number[a] of sunbathing occasions during the summer (April–September) | | | |
| None (*n* = 103) | 89 (83–95) | 8 (3–13) | 3 (0–6) |
| 1–14 (*n* = 294) | 67 (62–72) | 26 (20–30) | 8 (4–10) |
| ≥ 15 (*n* = 485) | 46 (42–51) | 38 (34–42) | 15 (12–19) |

[a] Average past and present number each summer.

estimate of number of times per year was calculated by multiplying number of times per week by number of weeks per year. In the same manner an estimate of total number of sunbed uses was calculated by multiplying number of times per year by number of years of regular use.

No re-examination of the histopathological slides was undertaken. However, all pathology reports were reviewed to ascertain that each case had a histopathologically confirmed diagnosis of *invasive* malignant melanoma. According to the pathology reports the diagnoses were superficial spreading melanoma in 309 cases (54%), nodular melanoma in 76 cases (13%), lentigo maligna melanoma in 57 cases (10%), acral lentiginous melanoma in three cases (0.5%) and unclassified invasive malignant melanoma in 113 cases (20%). Thirteen cases (2%) were incorrectly reported as invasive malignant melanoma, since they had the diagnosis of in situ melanoma.

Analyses were performed on histopathologically confirmed primary cutaneous malignant melanomas with the inclusion of 13 patients with in situ melanoma. Odds ratios (ORs) were computed, based on matched pairs, using both univariate and multivariate methods. In the multivariate analyses conditional logistic regression was used. The multivariate models included adjustments for hair colour, number of raised naevi, skin reaction to sun exposure (skin type) and number of sunburns, which were important risk factors identified in this case–control study as well as in our previous study (Westerdahl et al, 1994). The inclusions of other constitutional factors, other sun exposure variables and/or socio-economic factors into the multivariate models were found to give no further significant contribution to the chosen model. *P*-value less than 0.05 was considered statistically significant and 95% confidence intervals (CIs) were used. The statistical program Stata was utilized (Stata Corporation, College Station, TX, USA). Occasional missing values for some variables caused slight variations in the numbers of cases and controls used for each analysis. Cases and controls were not contacted to complement missing values. However, for almost all variables less than 5% were missing.

The Ethical Committee of the Medical Faculty of Lund University approved this study. Informed consent was sought from the treating physician, the patient and the healthy control.

### RESULTS

Among 571 cases (females, 50.3%; males, 49.7%) and 913 controls (females, 50.8%; males, 49.2%), 250 cases (44%) and 372 controls (41%) reported ever using sunbeds. Table 1 shows characteristics of the controls that had ever used sunbeds, separating the exposure ever use and regular use, past or present (hereafter referred to as regular use). As can be seen sunbed users reported more sunbathing occasions during the summer and had more raised naevi than non-sunbed users. Exposure to sunbeds was also seen to be more prevalent among younger persons and among females.

 *© 2000 Cancer Research Campaign*

**Table 2** Odds ratios (OR) for developing cutaneous malignant melanoma in relation to sunbed related variables among regular sunbed users

| Factor and category | Cases | Controls | OR (95% CI) | | OR-adj[a] (95% CI) | | Test for trend *P*-value |
|---|---|---|---|---|---|---|---|
| Exposure to sunbed | | | | | | | |
| Never | 319 | 538 | 1.0[b] | | 1.0[b] | | |
| Sometime | 162 | 270 | 1.1 | (0.8–1.4) | 1.1 | (0.8–1.4) | |
| Regular (present or past) | 88 | 102 | 1.6 | (1.1–2.4) | 1.8 | (1.2–2.7) | 0.05 |
| Number of sunbed uses per year | | | | | | | |
| None | 319 | 538 | 1.0[b] | | 1.0[b] | | |
| 1–5 | 9 | 16 | 0.8 | (0.3–2.6) | 1.5 | (0.3–6.4) | |
| 6–10 | 20 | 14 | 4.1 | (1.4–11.9) | 4.2 | (1.3–13.0) | |
| 11–15 | 14 | 17 | 2.2 | (0.8–5.8) | 2.7 | (1.0–8.3) | |
| ≥20 | 44 | 55 | 1.3 | (0.7–2.2) | 1.5 | (0.8–3.0) | 0.06 |
| Number of years of regular use | | | | | | | |
| None | 319 | 538 | 1.0[b] | | 1.0[b] | | |
| 1–10 | 58 | 65 | 2.1 | (1.2–3.7) | 2.7 | (1.4–5.4) | |
| >10 | 28 | 28 | 1.5 | (0.7–3.2) | 1.8 | (0.8–4.1) | 0.72 |
| Total number of sunbed uses | | | | | | | |
| None | 319 | 538 | 1.0[b] | | 1.0[b] | | |
| 1–125 | 22 | 32 | 1.7 | (0.8–3.7) | 2.8 | (1.0–7.8) | |
| 126–250 | 34 | 31 | 2.2 | (1.1–4.4) | 3.1 | (1.3–7.1) | |
| >250 | 31 | 37 | 1.3 | (0.7–2.5) | 1.5 | (0.7–3.2) | 0.26 |
| Age at first exposure | | | | | | | |
| Never | 319 | 538 | 1.0[b] | | 1.0[b] | | |
| ≤35 | 50 | 56 | 2.0 | (1.2–3.5) | 2.3 | (1.2–4.2) | |
| >35 | 38 | 44 | 1.6 | (0.9–2.5) | 1.6 | (0.9–2.9) | |
| Location of sunbed use[c] | | | | | | | |
| Never use | 319 | 538 | 1.0[b] | | 1.0[b] | | |
| At home | 34 | 38 | 1.3 | (0.7–2.5) | 1.5 | (0.7–3.3) | |
| Outside home | 52 | 64 | 1.6 | (0.9–2.8) | 2.2 | (1.1–4.4) | |
| Season when exposure took place[d] | | | | | | | |
| Never use | 319 | 538 | 1.0[b] | | 1.0[b] | | |
| Winter | 76 | 82 | 1.7 | (1.1–2.8) | 2.3 | (1.3–4.3) | |
| Summer | 4 | 9 | 0.9 | (0.2–3.1) | 0.5 | (0.1–2.7) | |
| Winter and summer | 8 | 11 | 1.1 | (0.4–3.4) | 1.4 | (0.4–4.8) | |

[a] Adjusted for hair colour, number of raised naevi, skin type and number of sunburns. [b]Reference category. [c]The place where sunbed exposure mainly take/took place. [d]The season when sunbed exposure mainly take/took place. Winter refers to October to March, while summer designates April to September.

The exposure to sunbeds was found to have greatly increased since the early 1980s, with 80% of such exposure among cases and 79% of such exposure among controls, being reported to have started after 1980. Ninety per cent of cases and 88% of controls, respectively, reported that they generally exposed the whole body for 30 min each time they used sunbeds.

The OR for developing malignant melanoma after ever having used sunbeds was 1.2 (95% CI 0.9–1.6), adjusted for history of sunburn after age 19 years, hair colour, skin type and number of raised naevi. In individuals younger than age 36, the OR was higher (adjusted OR = 3.1, 95% CI 0.7–13.4).

When all cases were considered, an elevated adjusted OR for the disease after regular exposure was found (adjusted OR = 1.8, 95% CI 1.2–2.7) (Table 2). Risk for melanoma was associated with number of sunbed uses per year, total number of times a sunbed was used and number of years of regular use. However, a dose–response relationship between total number of sunbed and risk of malignant melanoma was only found up to the level of 250 times. Above that level the elevated OR decreased and was not significant. Virtually the same pattern was seen for number of years of regular use and number of times per year respectively.

Interestingly, a higher risk was related to commercial use of sunbeds (Table 2). Regular use during the winter was also associated with an increased OR for melanoma development (adjusted OR = 2.3, 95% CI 1.3–4.3).

Regular sunbed users who were first exposed before age 36 demonstrated an increased OR for melanoma development (Table 2). Furthermore, in analyses of different age strata, melanoma patients younger than age 36 years showed the highest OR (adjusted OR = 8.1, 95% CI 1.3–49.5) for regular exposure to sunbeds vs. never (Table 3).

Adjusted ORs among men and women were 1.3 (95% CI 0.7–1.7) and 2.1 (95% CI 1.2–3.6), respectively, for regular vs. never use.

Individuals with black/dark brown or light brown hair had a higher and statistically significant adjusted OR than individuals with blond/fair or red hair (adjusted OR = 2.3, 95% CI 1.3–4.0, and adjusted OR = 1.5, 95% CI 0.2–10.4 respectively, for regular vs never use). The adjusted ORs for developing melanoma after regular sunbed use were almost the same among subjects who reported frequent sunbathing during the summer and those who did not sunbathe frequently during the summer (adjusted OR = 2.4, 95% CI 1.1–5.1 and adjusted OR = 3.0, 95% CI 0.8–10.8 respectively).

Analyses on sunbed use and risk of melanoma by histologic type showed similar ORs for the different histologic types, but the relation was statistically significant only for superficial spreading melanoma (adjusted OR = 1.8, 95% CI 1.0–3.3). In an analysis of exposure to sunbeds for subsites of melanoma (Table 4), the pattern of higher risk of melanoma among those with regular

© 2000 Cancer Research Campaign 

**Table 3** Odds ratios (OR) for developing cutaneous malignant melanoma in relation to regular use of sunbeds in different age[a] groups

| Factor and category | Cases | Controls | OR (95% CI) | | ORadj[b] (95% CI) | | Test for trend P-value |
|---|---|---|---|---|---|---|---|
| Patients younger than age 36 years | | | | | | | |
| Exposure to sunbed | | | | | | | |
| Never | 6 | 17 | 1.0[c] | | 1.0[c] | | |
| Sometime | 23 | 41 | 1.8 | (0.6–5.5) | 2.8 | (0.6–12.4) | |
| Regular (present or past) | 28 | 29 | 4.2 | (1.2–15.6) | 8.1 | (1.3–49.5) | 0.04 |
| Patients between ages 36 and 60 years | | | | | | | |
| Exposure to sunbed | | | | | | | |
| Never | 94 | 170 | 1.0[c] | | 1.0[c] | | |
| Sometime | 95 | 158 | 1.1 | (0.8–1.6) | 1.2 | (0.8–1.8) | |
| Regular (present or past) | 47 | 53 | 1.7 | (1.0–2.8) | 2.2 | (1.2–3.9) | 0.07 |
| Patients older than age 60 years | | | | | | | |
| Exposure to sunbed | | | | | | | |
| Never | 219 | 351 | 1.0[c] | | 1.0[c] | | |
| Sometime | 44 | 71 | 1.0 | (0.7–1.6) | 0.8 | (0.5–1.4) | |
| Regular (present or past) | 13 | 20 | 1.0 | (0.5–2.1) | 0.9 | (0.4–2.2) | 0.95 |

[a]Age at diagnosis. [b]Adjusted for hair colour, number of raised naevi, skin type and number of sunburns. [c]Reference category.

**Table 4** Odds ratios (ORs) and 95% confidence intervals (CI) for subgroups of melanoma by body site in relation to use of sunbeds

| Factor and category | Males | | Females | | Total (males + females) | |
|---|---|---|---|---|---|---|
| | OR adj[a] (95% CI) | Trend test P-value | OR adj[a] (95% CI) | Trend test P-value | OR adj[a] (95% CI) | Trend test P-value |
| Primary melanoma of the face and neck (39 males and 39 females) | | | | | | |
| Exposure to sunbed | | | | | | |
| Never | 1.0[b] | | 1.0[b] | 1.0[b] | | |
| Sometime | 0.2 (0.01–2.7) | 0.6 (0.1–2.7) | 0.4 (0.1–1.4) | | | |
| Regular | 0.8 (0.04–19.5) | 0.78 | 0.6 (0.1–5.6) | 0.05 | 0.5 (0.1–2.4) | 0.03 |
| Primary melanoma of the trunk (170 males and 96 females) | | | | | | |
| Exposure to sunbed | | | | | | |
| Never | 1.0[b] | | 1.0[b] | 1.0[b] | | |
| Sometime | 1.2 (0.7–2.2) | 0.7 (0.3–1.4) | 0.9 (0.6–1.4) | | | |
| Regular | 1.5 (0.5–4.4) | 0.02 | 1.6 (0.6–4.0) | 0.36 | 1.8 (0.9–3.3) | 0.06 |
| Primary melanoma of the upper extremities (41 males and 41 females) | | | | | | |
| Exposure to sunbed | | | | | | |
| Never | 1.0[b] | | 1.0[b] | 1.0[b] | | |
| Sometime | 1.0 (0.3–3.0) | 1.1 (0.2–5.7) | 0.8 (0.3–2.0) | | | |
| Regular | 0.9 (0.1–7.6) | 0.20 | 2.7 (0.3–21.5) | 0.02 | 1.6 (0.4–6.1) | 0.04 |
| Primary melanoma of the lower extremities (27 males and 109 females) | | | | | | |
| Exposure to sunbed | | | | | | |
| Never | 1.0[b] | | 1.0[b] | 1.0[b] | | |
| Sometime | 2.8 (0.4–8.2) | 1.7 (0.8–3.5) | 1.7 (0.9–3.1) | | | |
| Regular | 2.7 (0.2–20.0) | 0.11 | 2.7 (1.1–6.8) | 0.23 | 2.4 (1.1–5.7) | 0.14 |

[a]Adjusted for hair colour, number of raised naevi, skin type and number of sunburns. [b]Reference category.

sunbed use was seen for lesions of the trunk, upper and lower extremities. However, the latter association was the only one that reached statistical significance (adjusted OR = 2.1, 95% CI 1.1–4.2). When men and women were considered separately, lesions of the lower extremities showed the strongest associated with use of sunbeds in women (adjusted OR = 2.7, 95% CI 1.1–6.8) while no significant association was seen between sunbed use and anatomic site in men.

It is arguable that adjusting for naevi may be overmatching. However, adjusting for raised naevi did not appreciably affect the results, speaking against overmatching.

Analyses adjusted for other aspects of sun exposure gave virtually the same ORs. For instance, in a similar multivariate model as the one concerning 'Exposure to sunbed' in Table 2, the OR for developing melanoma after regular sunbed use was 1.8 (95% CI 1.2–2.7) when adjusting for sunbathing vacations abroad, and 1.9

© 2000 Cancer Research Campaign

(95% CI 1.2–2.8) when controlling for sunbathing frequency during the summer. In the same manner adjustments for other constitutional factors and/or socio-economic variables gave essentially the same results. Furthermore, when the 13 cases with in situ melanoma were excluded from the analyses the results were unaltered.

### DISCUSSION

The results of the present report point to a relation between sunbed use and malignant melanoma.

A very important issue when interpreting results on the association between use of sunbeds and melanoma development is the possibility of confounding by sun exposure. This potential confounding is of especial concern since it has been shown, like in the present study, that sunbed use correlate strongly with tanning in natural sunlight (Autier et al, 1991; Lillquist et al, 1994; Boldeman et al, 1997). Consequently the pattern of sunbed use might affect the risk of melanoma by the use per se, by being a surrogate for intermittent exposure to sunlight or by increasing the total UV dose received. In order to try separating the effect of sunbed use alone from that of sun exposure for the development of melanoma, the analyses were carefully adjusted for different sun exposure variables. However, the association between use of sunbed and malignant melanoma persisted after these adjustments. Even in analyses stratified by sunbathing habits the ORs turned out to be essentially the same.

A drawback of the present study is the lack of information on the types of lamp used and thus intensity and spectral outputs of the devices to which exposure had occurred. However, it is difficult, if not impossible, in a retrospective study design to collect detailed information on output spectra and intensity and at the same time expect high recall and minimum of memory bias. Furthermore, the nature of the sunlamps has changed over time, which makes it even more difficult. The lamps in use before the late 1970s produced significant fractions of UV-B (22–40%) and UV-C (0.1–20%) (Diffey and Farr, 1991). Since the early 1980s the devices produce mainly UV-A, but also a small fraction of UV-B (< 0.1–2.1%) (Diffey and Farr, 1991) since it produces a more substantial tan than UV-A. In recent years the fraction of UV-B produced by these devices has increased in Sweden (Swedish Radiation Protection Institute, personal communication). Yet, in the present study we do know that the tanning was for non-medical reason, that 80% of exposure started after 1980 and that approximately 90% had whole-body exposure for 30 min each time. We therefore believe that the subjects in this study were mostly exposed to devices mainly emitting UV-A.

The present results are in accordance with our previous results from South Sweden (Westerdahl et al, 1994), as well as results from Scotland (Swerdlow et al, 1988), Canada (Walter et al, 1990), and Belgium, France and Germany (Autier et al, 1994). All these studies have mainly come from areas with relatively low ambient UV radiation. They have all pointed to an association between use of sunbeds/sunlamps and malignant melanoma with some form of dose–response relation. Interestingly, in the present study this dose–response relationship only existed to a certain level above which the ORs decreased and became non-significant. Unfortunately the relationship between burns due to use of sunbeds and melanoma could not be assessed since only information on sunburns was recorded. Autier et al (1994) reported a strong association between burns due to sunlamp or sunbed exposure and melanoma.

Our results demonstrated higher risks both among those who started to use sunbeds earlier in life and among those diagnosed with melanoma at an earlier age. Previous studies have shown similar results (Chen et al, 1998; Swerdlow et al, 1988; Walter et al, 1990; Westerdahl et al, 1994). These findings may not be surprising since sunbed use is particularly common in teenagers and young adults (Boldeman et al, 1996) and the high OR might then be due to the high exposure in this age group per se. In addition, exposure at a younger age may have a greater impact on later melanoma development. Thus the lag period between onset of exposure and the occurrence of melanoma might not be that long after all.

To find out if the relation between sunbed use and risk for melanoma development differ according to sun sensitivity, a stratified analysis by hair colour was performed. A significantly increased odds ratio was only seen among subjects with black/dark brown or light brown hair. Additional subgroup analysis showed that an increased melanoma risk was associated with sunbed use outside home. Two previous studies have reported a higher risk with domestic use of sunbeds or sunlamps (Walter et al, 1990; Chen et al, 1998). However, others have suggested that the danger might be greater in the commercial sector because the output for both UV-A and UV-B are higher (Wright et al, 1997). There was a significant relation between exposure to sunbed during the winter and melanoma. This observation is most interesting since it is reasonable to presume that during the winter the skin of most individuals in our country is less adapted to UV radiation than in the summer.

In contrast to our previous study (Westerdahl et al, 1994) lesions of the legs were those that showed the strongest association of increased risk with sunbed use. The use of sunbeds was only borderline significantly associated with lesions of the trunk. This observation is most interesting since exposure to sunbeds was seen to be more prevalent among females. It was associated with increased risk for developing melanoma in females but not in men. Melanomas appear most frequently on the lower limbs in females. Finally, the age-standardized incidence rates have particularly increased for cutaneous malignant melanoma of the trunk in both men and women and of the leg in women (Østerlind et al, 1988*a*; Thörn et al, 1990). Walter et al (1990) found that the sunbed-related risk was greater for men and for melanoma of the face/head/neck and arms.

Five additional studies have reported limited (Chen et al, 1998) or no evidence of an association between sunlamp/sunbed use and malignant melanoma (Gallagher et al, 1986; Holman et al, 1986; Østerlind et al, 1988*b*; MacKie et al, 1989). However, most of these studies were based on relatively small number of subjects who were exposed to sunbeds and have presented very limited information on sunbed use. The present study provides more detailed exposure data.

In addition to the epidemiological evidence presented, it seems biologically plausible that exposure to sunbeds could increase the risk of melanoma since UV-A, like UV-B, has been classified as ‘probably carcinogenic to humans’ (IARC, 1992). A recent study has also shown a significant increase in risk of cutaneous melanoma among patients treated with oral psoralen and UV-A radiation (Stern et al, 1997). However, the study by Stern and co-workers did not report the total phototoxic dose delivered to their patients or if the melanoma patients were at greater risk for

*© 2000 Cancer Research Campaign*

melanoma in the first place (Wolff, 1997). Furthermore, it has been suggested that UV-A sunbeds may cause melanocytic lesions with malignant potential (Jones et al, 1987; Williams et al, 1988). Lastly, the only existing animal model of melanoma for which an action spectrum has been estimated, the platyfish-swordtail hybrid model, shows that UV-A exposure may be more important than UV-B (Setlow et al, 1993). Indeed, in terms of biologically effective doses (applying action spectra for the platyfish-swordtail hybrid model), frequent sunbed use can increase the annual effective dose as much as six times what would be received from the sun alone (Miller et al, 1998). When considering all these data together, it may be tempting to suggest that primarily UV-A and not UV-B is associated with the development of melanoma. This is in accordance with a new hypothesis for melanoma induction, which proposes that radiation absorbed by the melanin in melanocytes generates products that may activate the carcinogenic process (Moan et al, 1999). This is thought to be true especially for UV-A radiation since it penetrates the skin deeper and better than UV-B radiation.

To reduce the likelihood of selection bias, the study had a population-based design. Furthermore, the response rate was reasonably good. There is also no a priori reason to suspect that identified risk factors in this study are associated with non-participation.

In order to reduce measurement errors attention was paid to defining variables in such a way that one could expect high recall with minimum of memory bias. Indeed, a similar questionnaire has been found to yield information with good test–retest reliability (Westerdahl et al, 1996). Still, the influence of non-differential misclassification (i.e. measurement error that is independent of disease status) may have been considerable, since for instance the exposure variable sunbed use most likely included a heterogeneous sample of devices. However, it is widely appreciated that non-differential misclassification leads to an underestimation of a true relationship. A particular concern in case–control studies is recall bias (i.e. if cases report differently than controls), since it can distort associations in an unpredictable manner (Copeland et al, 1977). We used identical procedures of data collection for cases and controls. In addition, information from cases was collected close in time to the diagnosis in order to avoid the influence, which the diagnosis of melanoma may have on recall of sunbed use. Nevertheless, it can not be solely ruled out that awareness of the diagnosis of malignant melanoma and the hypothesis of an association between sunbed use and melanoma occurrence may have perverted the answers to the questions on sunbed use. However, in the present study the estimated risks were virtually the same as those obtained when the general population was unaware of the hypothesis (Westerdahl et al, 1994). Moreover, a higher rate of both cases and controls reported exposure to sunbeds in the present study (cases: 44%; controls: 41%) compared to our previous study (cases: 29%; controls: 24%). We therefore do not think that reporting errors differs between cases and controls.

In conclusion, although it is not possible to entirely rule out that the observed relationship may be partly explained by residual confounding of inadequate measurement of sun exposure the present results substantiate the hypothesis that exposure to sunbeds might increase the risk of developing malignant melanoma. Such relationship is of serious concern because of the widespread use of sunbeds in the modern societies. Our results are in accordance with the assumption that UV-A may be more important than UV-B for melanoma induction.

### ACKNOWLEDGEMENT

The present study was supported by the Swedish Cancer Society.

### REFERENCES

Autier P, Joarlette M, Lejeune F, Lienard D, Andre J and Achten G (1991) Cutaneous malignant melanoma and exposure to sunlamps and sunbeds: a descriptive study in Belgium. *Melanoma Res* **1**: 69–74

Autier P, Dore JF, Lejeune F, Koelmel KF, Geffeler O, Hille P, Cesarini JP, Lienard D, Liabeuf A, Joarlette M, Chemaly P, Hakim K, Koeln A and Kleeberg UR (1994) Cutaneous malignant melanoma and exposure to sunlamps or sunbeds: an EORTC multicenter case–control study in Belgium, France and Germany. EORTC Melanoma Cooperative Group. *Int J Cancer* **58**: 809–813

Boldeman C, Beitner H, Jansson B, Nilsson B and Ullén H (1996) Sunbed use in relation to phenotype, erythema, sunscreen use and skin diseases. A questionnaire survey among Swedish adolescents. *Br J Dermatol* **135**: 712–716

Boldeman C, Jansson B, Nilsson B and Ullén H (1997) Sunbed use in Swedish urban adolescents related to behavioral characteristics. *Prev Med* **26**: 114–119

Chen YT, Dubrow R, Zheng T, Barnhill RL, Fine J and Berwick M (1998) Sunlamp use and the risk of cutaneous malignant melanoma: a population-based case–control study in Connecticut, USA. *Int J Epidemiol* **27**: 758–765

Copeland KT, Checkoway H, McMichael AJ and Holbrook RH (1977) Bias due to misclassification in the estimation of relative risk. *Am J Epidemiol* **105**: 488–495

Diffey BL and Farr PM (1991) Tanning with UVB or UVA: an appraisal of risks. *J Photochem Photobiol B* **8**: 219–223

Gallagher RP, Elwood JM and Hill GB (1986) Risk factors for cutaneous malignant melanoma: the Western Canada Melanoma Study. *Recent Results Cancer Res* **102**: 38–55

Holman CD, Armstrong BK, Heenan PJ, Blackwell JB, Cumming FJ, English DR, Holland S, Kelsall GR, Matz LR, Rouse IL, Singh A, Ten Seldam REJ, Watt JD and Xu Z (1986) The causes of malignant melanoma: results from the West Australian Lions Melanoma Research Project. *Recent Results Cancer Res* **102**: 18–37

IARC (1992) *IARC Monographs on the Evaluation of Carcinogenic Risks to Humans. Solar and Ultraviolet Radiation*. *IARC* Monogr *Eval Carcinog Risks Hum* **55**: 1–316

Jones SK, Moseley H and Mackie RM (1987) UVA-induced melanocytic lesions. *Br J Dermatol* **117**: 111–115

Lillquist PP, Baptiste MS, Witzigman MA and Nasca PC (1994) A population-based survey of sun lamp and tanning parlor use in New York State, 1990. *J Am Acad Dermatol* **31**: 510–512

MacKie RM, Freudenberger T and Aitchison TC (1989) Personal risk-factor chart for cutaneous melanoma [see comments]. *Lancet* **2**: 487–490

Miller SA, Hamilton SL, Wester UG and Cyr WH (1998) An analysis of UVA emissions from sunlamps and the potential importance for melanoma. *Photochem Photobiol* **68**: 63–70

Moan J, Dahlback A and Setlow RB (1999) Epidemiological support for an hypothesis for melanoma induction indicating a role for UVA radiation. *Photochem Photobiol* **70**: 243–247

Østerlind A, Engholm G and Jensen OM (1988*a*) Trends in cutaneous malignant melanoma in Denmark 1943–1982 by anatomic site. *APMIS* **96**: 953–963

Østerlind A, Tucker MA, Stone BJ and Jensen OM (1988*b*) The Danish case–control study of cutaneous malignant melanoma. II. Importance of UV-light exposure. *Int J Cancer* **42**: 319–324

Setlow RB, Grist E, Thompson K and Woodhead AD (1993) Wavelengths effective in induction of malignant melanoma. *Proc Natl Acad Sci USA* **90**: 6666–6670

Stern RS, Nichols KT and Vakeva LH (1997) Malignant melanoma in patients treated for psoriasis with methoxsalen (psoralen) and ultraviolet A radiation (PUVA). The PUVA Follow-Up Study [see comments]. *N Engl J Med* **336**: 1041–1045

Swerdlow AJ, English JS, MacKie RM, O'Doherty CJ, Hunter JA, Clark J and Hole DJ (1988) Fluorescent lights, ultraviolet lamps, and risk of cutaneous melanoma [published erratum appears in *Br Med J* 1988 **297**(6657): 1172]. *Br Med J* **297**: 647–650

 *© 2000 Cancer Research Campaign*

Thörn M, Bergström R, Adami HO and Ringborg U (1990) Trends in the incidence of malignant melanoma in Sweden, by anatomic site, 1960–1984. *Am J Epidemiol* **132**: 1066–1077

Walter SD, Marrett LD, From L, Hertzman C, Shannon HS and Roy P (1990) The association of cutaneous malignant melanoma with the use of sunbeds and sunlamps. *Am J Epidemiol* **131**: 232–243

Westerdahl J, Olsson H, Måsbäck A, Ingvar C, Jonsson N, Brandt L, Jönsson PE and Möller T (1994) Use of sunbeds or sunlamps and malignant melanoma in southern Sweden. *Am J Epidemiol* **140**: 691–699

Westerdahl J, Anderson H, Olsson H and Ingvar C (1996) Reproducibility of a self-administered questionnaire for assessment of melanoma risk. *Int J Epidemiol* **25**: 245–251

Williams HC, Salisbury J, Brett J and du Vivier A (1988) Sunbed lentigines. *Br Med J (Clin Res Ed)* **296**: 1097

Wolff K (1997) Should PUVA be abandoned? [editorial; comment]. *N Engl J Med* **336**: 1090–1091

Wright A, Hart G and Kernohan L (1997) Dangers of sunbeds are greater in the commercial sector [letter; comment]. *Br Med J* **314**: 1280–1281

*© 2000 Cancer Research Campaign*
